# Development and Evaluation of “a PEGylated Anti-Tau ScFv for SPECT Imaging” in a Rat Model of Traumatic Brain Injury

**DOI:** 10.3390/pharmaceutics18050626

**Published:** 2026-05-20

**Authors:** Esmat Sajjadi, Ehsan Sharif-Paghaleh, Mohammad Akrami, Koorosh Shahpasand, Ismaeil Haririan, Samane Maghsoudian

**Affiliations:** 1 Department of Pharmaceutical Biomaterials and Medical Biomaterials Research Center, Faculty of Pharmacy, Tehran University of Medical Sciences, 16Azar Street, Tehran 1417614411, Iran; esmatsajadi11@gmail.com; 2 Department of Immunology, School of Medicine, Tehran University of Medical Sciences, Tehran 1461884513, Iran; e-sharif@tums.ac.ir; 3 Department of Imaging Chemistry and Biology, School of Biomedical Engineering and Imaging Sciences, Faculty of Life Sciences and Medicine, King’s College London, London SE1 7EH, UK; 4 Department of Laboratory Medicine and Pathology, University of Minnesota Medical School, Minneapolis, MN 55414, USA; shahpasand@royaninstitute.org; 5 Department of Pharmaceutics, Faculty of Pharmacy, Tehran University of Medical Sciences, Tehran 1417614411, Iran; 6 Department of Pharmaceutical Nanotechnology, Faculty of Pharmacy, Tehran University of Medical Sciences, Tehran 1417614411, Iran; s-maghsoudian@alumnus.tums.ac.ir

**Keywords:** PEGylation, 99m Tc labeled scFv, Tau-targeted SPECT imaging, TBI, neuroimaging

## Abstract

**Background:** Traumatic brain injury (TBI) affects millions of individuals annually and remains a major global cause of neurological disability and death. Tau protein hyperphosphorylation, particularly in its cis conformation, is a major pathological hallmark contributing to neurodegeneration following TBI. Single-chain variable fragments (scFvs), despite their diagnostic potential, suffer from rapid renal clearance and short circulation half-lives, which limit their in vivo performance. PEGylation is therefore employed to prolong systemic circulation and improve the pharmacokinetic behavior of scFvs, enabling more effective brain retention and target engagement. **Methods:** In this study, we utilized a previously validated anti-cis p-tau scFv antibody fragment, radiolabeled with technetium-99m tricarbonyl (^99m^Tc(CO)_3_), as a diagnostic tracer to detect tau pathology in TBI rat models. The antibody was conjugated with polyethylene glycol (PEG, 20 kDa); PEGylation efficiency was determined by quantifying the products on SDS-PAGE, and the products were subsequently radiolabeled. **Results:** Radiochemical purity (RCP) was ~95.4% for the non-PEGylated tracer (^99m^Tc-AININ20) and ~92.7% for the PEGylated form (^99m^Tc-AININ20-PEG), with both showing >90% radiochemical purity consistently. Upon systemic administration, PEGylated scFv was able to cross the blood–brain barrier (BBB) and selectively accumulated in injured regions, as confirmed by single-photon emission computed tomography (SPECT) imaging. Both PEGylated and non-PEGylated scFv tracers showed significantly higher brain uptake in TBI rats compared to healthy controls (*p* < 0.0001). At 24 h, the PEGylated form exhibited a significantly higher brain signal than the non-PEGylated version (*p* < 0.0001), indicating improved tracer retention. Biodistribution analysis at 2 h post-injection showed significantly reduced renal clearance for the PEGylated tracer and increased hepatic uptake compared to the non-PEGylated form. At 24 h, in vivo imaging confirmed sustained brain retention, highlighting improved pharmacokinetics and imaging potential. **Conclusions:** These results support PEGylated scFv as a promising SPECT imaging agent for early detection of tauopathy in TBI, offering enhanced brain retention and improved pharmacokinetics.

## 1. Introduction

Traumatic brain injury (TBI) is a disruption in normal brain function caused by an external force, such as a blow or jolt to the head [1,2,3]. According to the World Health Organization (WHO), TBI is projected to become the third leading cause of death and disability worldwide by 2030 [4], reflecting its growing burden on global health. In 2019, there were approximately 27.16 million new cases of TBI globally, with a total of 48.99 million individuals living with TBI-related disabilities [5]. Chronic TBI refers to the long-term sequelae of brain trauma, which often result in persistent cognitive, emotional, and physical impairments [6,7]. Individuals with chronic TBI are at elevated risk of developing neurodegenerative diseases such as Alzheimer’s disease (AD) [8,9,10,11] and chronic traumatic encephalopathy (CTE), sometimes decades after the initial insult [12,13].

This increased vulnerability is attributed to the accumulation of pathological proteins, particularly hyperphosphorylated tau [14,15,16], which contributes to progressive neurodegeneration [17]. Among various phosphorylation sites, tau phosphorylation at threonine 231 (p231)—especially in its cis conformation—is a key early and neurotoxic event. This conformer disrupts microtubule stability and induces neuronal death, making cis-pT231 tau a promising biomarker for early diagnosis of tauopathies.

Given the cascading molecular events following TBI, there is a critical need for early detection strategies to identify and monitor tau pathology [18,19,20]. Advanced imaging techniques such as magnetic resonance imaging (MRI) [21,22], positron emission tomography (PET) [23,24,25], and single-photon emission computed tomography (SPECT) [26,27,28] provide tools to detect structural and molecular changes post-TBI. In particular, PET-based imaging of pathological protein aggregates has been extensively investigated and clinically validated, while also highlighting challenges related to signal interpretation and quantitative assessment [25,29]. SPECT imaging with radiolabeled agents targeting tau protein offers a non-invasive approach to visualize disease progression and detect early tau pathology. In this case, radiolabeled antibody fragments [30,31,32] have shown potential as molecular imaging agents in preclinical studies. Compared to full antibodies, single-chain variable fragments (scFvs) offer superior tissue penetration and faster clearance, reducing background signal and enhancing imaging contrast. Furthermore, these fragments can be easily labeled using technetium-99m tricarbonyl (^99m^Tc(CO)_3_), a widely available isotope with a suitable 6 h half-life and 140 keV gamma emission, optimal for SPECT and superior to ^111^In, ^123^I, or ^67^Ga [33]. The use of a polyhistidine tag enables direct labeling under mild conditions, making ^99m^Tc a practical choice for preclinical molecular imaging studies [34,35,36]. However, scFvs suffer from rapid renal clearance and short circulation half-lives [37,38,39].

To address this limitation, PEGylation—the conjugation of polyethylene glycol (PEG) —is employed to enhance solubility, prolong systemic circulation, and improve pharmacokinetic profiles [40,41,42,43]. This modification improves the pharmacokinetic properties [44,45] and stability of scFvs, making them more effective for both diagnostic and therapeutic applications [46]. Previous studies have also shown that the lifetime of antibody fragments can be optimized by attaching polyethylene glycol chains (PEGs) to them [47].

Despite advances in neuroimaging, existing tau tracers such as ^18^F-flortaucipir (Tauvid) primarily detect mature neurofibrillary tangles, which form late in disease [48,49,50]. These tracers are inadequate for visualizing early pathological conformers like cis-pT231 tau [51,52]. Moreover, common SPECT tracers such as ^99m^Tc-HMPAO and ^99m^Tc-ECD also fall short, as they indirectly assess perfusion rather than directly targeting tau [53,54,55,56]. These limitations highlight the unmet need for an imaging agent to directly and sensitively target early tau pathology [52,57], before irreversible neuronal loss occurs. To address this unmet need, we employed AININ20, a previously developed scFv antibody that specifically binds to cis-pT231 tau [58]. In this study, we performed PEGylation of AININ20 to improve its pharmacokinetic properties and radiolabeled it with ^99m^Tc(CO)_3_. We evaluated both PEGylated and non-PEGylated forms in a rat model of TBI using SPECT imaging to assess brain uptake, tracer retention, and biodistribution. Our goal was to determine whether PEG-AININ20 serves as an effective and early diagnostic tool for tauopathy following TBI.

## 2. Methods and Materials

### 2.1. Materials

All solvents were purchased from Merck Company(Darmstadt, Germany). Chemicals and reagents were obtained from Sigma-Aldrich (St. Louis, MO, USA). The monoclonal anti-tau antibody (AININ20) was kindly provided as a gift from the Royan Institute (Tehran, Iran). Its sequence and generation are described in US Patent 10,570,195 B2 (SEQ ID NO:1–8). PEG-NHS was purchased from Jenkem Technology (Beijing, China). Amicon^®^ Ultra centrifugal filters (MWCO: 30 kDa) were purchased from Merck Millipore. Pertechnetate solution (^99m^TcO_4_^−^) was supplied by Pars Isotope Company (Tehran, Iran), and the technetium tricarbonyl kit was freshly prepared in-house. Additionally, the materials for SDS-PAGE analysis were mainly provided by the Merck company.

### 2.2. Immunofluorescence Staining of Rat Brain Sections

Immunofluorescence staining was performed on brain sections from TBI-induced adult male Wistar rats. Brain tissue sections were deparaffinized, rehydrated through graded ethanol, and treated with 0.3% hydrogen peroxide. Antigen retrieval was performed by briefly boiling the sections in 10 mM sodium citrate buffer (pH 6.0). After blocking with the manufacturer-supplied blocking buffer (ReadyProbes™, Invitrogen, Waltham, MA, USA), sections were incubated overnight at 4 °C with the primary antibody, cis anti-tau monoclonal antibody. The following day, sections were incubated with Alexa Fluor 488 or 568 secondary antibodies (Jackson ImmunoResearch, West Grove, PA, USA) for 1 h at room temperature. All steps were separated by thorough washing with TBS. Finally, sections were mounted using an antifade medium. The stained sections were visualized using a high-resolution fluorescent microscope (Olympus, Tokyo, Japan, BX51, equipped with an Olympus DP72 digital camera), which allowed for detailed observation of the specific localization and intensity of fluorescence, enabling accurate assessment of the targeted proteins within the tissue samples. Finally, the acquired images were analyzed using ImageJ software (version 1.54p).

### 2.3. Pegylation of Scfv

The PEGylation protocol employed in this study was adapted from the Thermo Scientific guidelines for NHS-activated PEG conjugation of proteins. Briefly, the reaction was carried out using methoxy-PEG-NHS ester (JenKem Technology, MW 20 kDa) in the phosphate-buffered saline (PBS) at pH ~7.4. The optimized reaction was performed at a PEG NHS to scFv molar ratio of [5:1], with a final scFv concentration of 1 mg/mL. The reaction mixture was incubated at 37 °C for 90 min with gentle mixing. Different molar ratios of PEG relative to the antibody were initially evaluated to determine the optimal conjugation conditions and ensure reproducibility [47,59]. Following the PEGylation reaction, unreacted PEG-NHS and unconjugated antibodies were removed through centrifuging the reaction mixture at 5000× *g* for 5 min through Amicon^®^ Ultra centrifugal filters (MWCO: 30 kDa), followed by three washes with approximately 100 µL of buffer (pH 7.4) each time.

### 2.4. Sds-Page Analysis

Pegylation efficiency of scFv was monitored using SDS-PAGE, conducted with a 12% running gel. Following electrophoresis, the gels were stained with a silver stain to visualize protein bands [47,60,61]. The stained gels were rinsed with distilled water and scanned for documentation. Molecular weight standards were run alongside the samples to estimate the protein sizes. Additionally, the intensity of the protein bands was quantified using ImageJ software to calculate the reaction yield based on the SDS-PAGE results.

### 2.5. Binding Assay

The binding affinity and specificity of the anti-cis-pT231-tau antibody before and after PEGylation were evaluated using an enzyme-linked immunosorbent assay (ELISA). High-binding 96-well polystyrene plates (Nunc MaxiSorp) were coated overnight at 4 °C with 100 µL of synthetic cis-pT231-tau peptide (5 µg/mL) in carbonate-bicarbonate buffer (pH 9.6). Wells were subsequently blocked with 5% bovine serum albumin (BSA) in PBS containing 0.05% Tween-20 for 1 h at room temperature. Serial dilutions of PEGylated and non-PEGylated antibodies in blocking buffer were added and incubated for 2 h at room temperature with gentle shaking. After three washes with PBS-T, HRP-conjugated secondary antibody (1:5000) was applied for 1 h. Wells were washed again, followed by TMB substrate solution (3,3′,5,5′-tetramethylbenzidine) for colorimetric detection. The reaction was stopped with 2N sulfuric acid, and absorbance was measured at 450 nm using a microplate reader (BioTek Synergy H1, Winooski, VT, USA). Binding curves were generated by plotting absorbance against antibody concentration to compare the affinities of PEGylated and non-PEGylated antibodies.

### 2.6. Radiolabeling Methods

#### 2.6.1. Preparation of [^99m^Tc(H_2_O)_3_(CO)_3_] ^+^ Precursor

**Figure 1:**
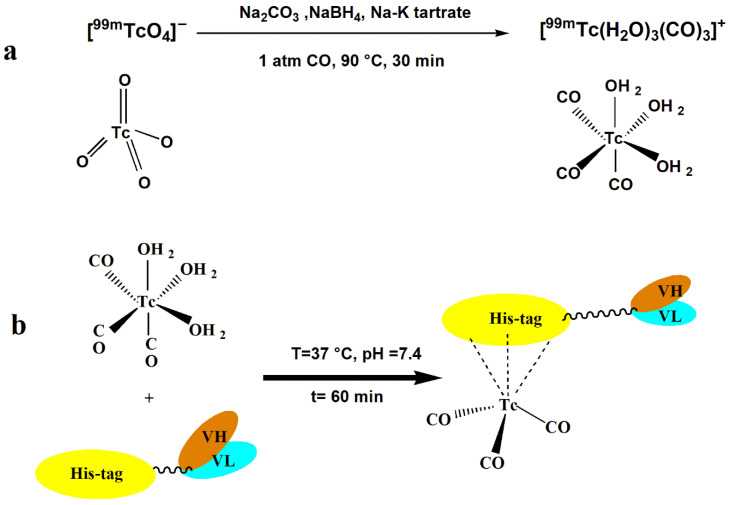
(**a**) Synthesis of [^99m^Tc(H_2_O)_3_(CO)_3_]^+^ precursor. (**b**) Radiolabeling of the scFv with the [^99m^Tc(H_2_O)_3_(CO)_3_]^+^ complex.

Preparation of the [^99m^Tc(H_2_O)_3_(CO)_3_]^+^ precursor was performed following the methodology outlined in the prior publication [62]. In brief, the fresh [^99m^TcO_4_]^-^ was conducted from the ^99^Mo/^99m^Tc generator using sterile saline. The eluted solution (~30 mCi, 1 mL) was introduced into a vial containing a mixture of NaBH_4_ (5.5 mg), Na_2_CO_3_ (4 mg), and Na-K tartrate (15 mg), sealed, and flushed with CO for 15 min. Subsequently, the vial was heated at 95 °C for 30 min (Figure 1a). Following cooling to room temperature, the pH of the solution was adjusted to approximately 7 using 1 M HCl. The radiochemical purity (RCP) of the resulting complex, [^99m^Tc(CO)_3_]^+^, was assessed using the Radio-thin Layer Chromatography (RTLC) technique [63], employing glass-backed silica gel 60 ITLC plates (Merck, Darmstadt, Germany) with a mobile phase of Methanol: HCl (95/5) [64], and analyzed using a gamma-ray TLC scanner (RTLC2118, CFP Co., Tehran, Iran). The purity was expressed as the ratio of the peak integral of [^99m^Tc(CO)_3_]^+^ (at R_f_ = 0.2–0.8) to the integral of the entire chromatogram, which includes peaks for ^99m^Tc colloids and unreduced [^99m^TcO_4_]^−^ at R_f_ = 0 and 0.9, respectively.

#### 2.6.2. Radiolabeling of Scfv with [^99m^Tc(H_2_O)_3_(CO)_3_]^+^ Precursor

The labeling was performed by incubating each antibody (100 µL, 1 mg/mL) with 100 µL of [^99m^Tc(H_2_O)_3_(CO)_3_]^+^ (~140 MBq) at 37 °C for 60 min, yielding high-purity tracers in both cases The radiolabeling protocol for both AININ20 and PEG-AININ20 was designed in this study based on previously published procedure [65,66,67]. In the radiolabeling process, each of them was incubated with [^99m^Tc(H_2_O)_3_(CO)_3_]^+^ (100 µL, ~140 MBq) at a temperature of 37 °C for 60 min, resulting in the production of high-purity tracers in both preparations (Figure 1). The final RCP of the samples was evaluated using ITLC with acetate buffer (pH 5.5) as a mobile phase. Analysis of the samples was performed using a gamma-ray TLC scanner (R_f_ proteins = 0, R_f_ [^99m^Tc(H_2_O)_3_(CO)_3_]^+^ = 1).

### 2.7. Stability Study

#### 2.7.1. Stability in Saline

To evaluate the stability of the labeled compounds, 100 µL of each of ^99m^Tc-AININ20 or ^99m^Tc-AININ20-PEG was incubated with 900 µL of saline at 37 °C. The stability of both compounds was monitored at various time points (0, 2, 4, 6, 8, and 24 h) using radio-TLC analysis.

#### 2.7.2. Stability in Human Serum

Human serum samples were obtained from healthy donors after written informed consent. Similarly, 100 µL of each of ^99m^Tc-AININ20 or ^99m^Tc-AININ20-PEG was mixed with 900 µL of freshly collected human serum and incubated at 37 °C. At selected time points (0, 1, 2, 4, 6, 8, and 24 h), samples were withdrawn and mixed with an equal volume of absolute ethanol to precipitate proteins. After centrifugation (4000 rpm, 5 min, 37 °C), the supernatant was analyzed by radio-TLC scanner to determine the radiochemical purity.

### 2.8. Estimation of Logp

The partition coefficient (LogP) of the radiocompounds in n-Octanol/PBS (pH 7.4) was assessed using an established procedure from the literature [68,69]. Approximately 100 µL of each labeled compound was separately mixed with 1 mL of phosphate-buffered saline (PBS, pH 7.4) and 1 mL of n-octanol in a micro-centrifuge tube. The mixture was vigorously vortexed for 10 min to ensure adequate mixing, followed by centrifugation at 1500 rpm for 4 min to achieve phase separation. Equal aliquots (100 µL) from each phase were collected, and the radioactivity was measured using a dose calibrator (CURIEMENTOR^®^ 4, PTW, Freiburg, Germany) in triplicate. LogP was calculated based on the equation:

LogP: LogP = Log (C Octanol/C PBS)(1)

### 2.9. In Vivo Study

All animal experiments and the use of human blood samples were conducted in accordance with institutional and national guidelines and approved by the Ethics Committee of Tehran University of Medical Sciences (Ethical code: IR.TUMS.TIPS.RES.1399.075).

#### 2.9.1. Animal Model

Adult male Wistar rats (10–12 weeks old, weighing 220–250 g) were housed under standard laboratory conditions, including a 12 h light/dark cycle, controlled temperature (22 ± 2 °C), and relative humidity (50 ± 10%), with free access to food and water. Animals were anesthetized via intraperitoneal injection of ketamine/xylazine (100 mg/kg to 10 mg/kg), and TBI was induced by dropping a 450 g weight from a height of 2 m, as previously described [70,71]. In the present study, double-immunofluorescence staining revealed a marked increase in pTau231 in the injured brain tissue (Supplementary Figure S1). Neuronal and axonal structures were labeled with MAP2/SMI-312, and nuclei were stained with DAPI. The strong pTau231 signal co-localizing with neuronal structures indicated abnormal tau phosphorylation and early tauopathy formation following TBI, consistent with previously reported TBI-induced tauopathy patterns [72,73,74]. The intravenous administration was performed by injecting 100 µL of each sample solution (1 mg/mL, 74 MBq) with 100 µL of normal saline via the tail vein.

For the imaging study, five experimental groups were used, with three rats per group. These groups were imaged up to 2 h post-injection at various time points (15, 30 min, 1, 2 h): (1) healthy rats receiving ^99m^Tc-AININ20, (2) TBI rats receiving ^99m^Tc-AININ 20, and (3) TBI rats treated with [^99m^Tc(H_2_O)_3_(CO)_3_]^+^. In contrast, two additional groups of TBI rats were monitored up to 24 h post-injection: one group received ^99m^Tc-AININ20, and the other received ^99m^Tc-AININ20-PEG. At the 24 h time point, these animals underwent in vivo SPECT imaging with a focus on the liver and kidneys to assess tracer retention and clearance through the major excretory organs. These imaging protocols were designed to compare the effects of brain injury and PEGylation on tracer biodistribution and retention.

#### 2.9.2. In Vivo Spect Imaging

SPECT imaging was performed using a small-animal micro-SPECT (Pars Negar Persia Co., Tehran, Iran). During imaging, rats were maintained under isoflurane anesthesia with continuous respiratory monitoring, and a heating pad was used to keep body temperature stable at 37 °C, as described previously [36,75]. Imaging studies were conducted 48 h following the induction of TBI. In all experimental groups, rats received an intravenous injection of 100 μL of the radiotracer (1 mg/mL, 74 MBq) diluted in 100 μL of saline. SPECT images were acquired at 10 and 30 min and 1, 3, 6, and 24 h post-injection to assess in vivo biodistribution and brain uptake. Image reconstruction and quantitative analysis were performed using VivoQuant software (version 3.0).

#### 2.9.3. Biodistribution Studies

The biodistribution of radiolabeled compounds was investigated in three groups of male Wistar rats (*n* = 3 per group), two hours post-injection: healthy controls, TBI models treated with native antibody (^99m^Tc-AININ20), and TBI models treated with PEGylated antibody (^99m^Tc-AININ20-PEG). Each rat received an intravenous injection via the tail vein of 100 µL of the radiolabeled antibody solution (1 mg/mL, 74 MBq), diluted in 100 µL of 0.9% sterile saline. After a 2 h post-injection period, animals were euthanized by Ketamine (100 mg/kg) and Xylazine (10 mg/kg), and pertinent organs were collected, weighed, and analyzed using a dose calibrator to determine the percentage of injected dose per gram (%ID/g) in each organ.

After a 2 h post-injection period, animals were euthanized using ketamine (100 mg/kg) and xylazine (10 mg/kg). The relevant organs were then excised, thoroughly perfused, and washed with normal saline to remove residual blood, gently blotted dry, weighed, and subsequently analyzed using a dose calibrator to determine the percentage of injected dose per gram (%ID/g) in each organ. The biodistribution was expressed as the mean standard deviation (Mean ± SD) for each organ, with a sample size of *n* = 3.

### 2.10. Statistical Analysis

Statistical analyses were conducted using 2-way ANOVA with appropriate post hoc multiple-comparisons tests (Tukey or Sidak). GraphPad Prism version 8.0 (GraphPad Software) was used for all statistical analyses and graph creation. Data are presented as mean ± standard deviation (SD); *p* values of 0.05 or less were considered significant.

## 3. Results and Discussion

In light of the growing concern over the long-term consequences of TBI, including its association with neurodegenerative diseases, early detection of tau pathology has become a critical focus [76]. Our study utilized PEGylated and non-PEGylated forms of ^99m^Tc-labeled AININ20, an antibody that selectively binds to cis-pT231 tau, as a molecular imaging probe in a rat model of TBI. This approach aimed to assess the potential of PEGylation in improving the pharmacokinetic properties of the scFv antibody, thereby improving its brain retention and diagnostic efficacy for early-stage tauopathies.

### 3.1. Pegylation of Scfv and Sds-Page Analysis

**Figure 2:**
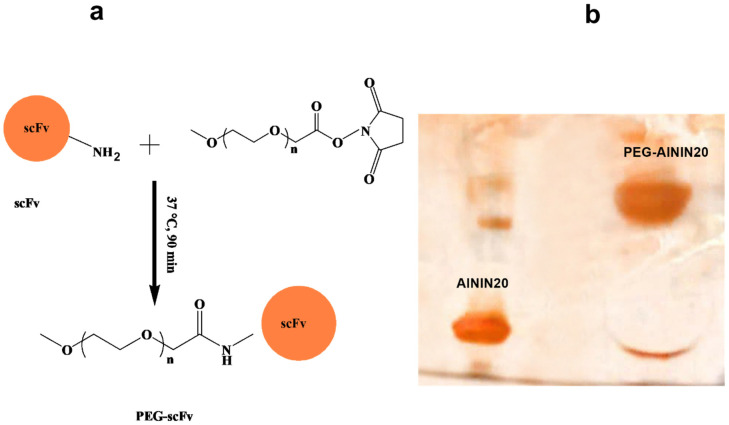
(**a**) Schematic illustration of the PEGylation reaction, showing the covalent attachment of PEG chains to the antibody via NHS-ester chemistry targeting lysine residues. (**b**) SDS-PAGE analysis of the antibody before and after PEGylation. The left lane shows the non-PEGylated antibody, and the right lane shows the PEGylated antibody. The observed shift to a higher molecular weight band confirms successful PEG conjugation.

PEGylation was performed using the PEG-NHS (20 kDa) with anti-tau scFv. Different molar ratios of PEG-NHS were selected for the reaction with scFv to optimize conjugation efficiency with Lys residues on the antibody surface, as described in Section 2.3. Following the reaction, the mixture was purified by centrifugal filtration (Amicon 30 kDa) by washing it against PBS to remove unreacted PEG and scFv. The purified products were then analyzed by SDS-PAGE (12% gel), which confirmed successful PEGylation through the appearance of a band with higher molecular weight compared to the non-PEGylated scFv visualized with silver nitrate staining (Figure 2) [47,77]. PEGylation efficiency was determined using ImageJ software based on band intensities. Since PEGylation was performed in a site-specific manner at a single conjugation site, the reaction is expected to predominantly produce a mono-PEGylated species, which is consistent with the observation of a single dominant higher–molecular–weight band in SDS–PAGE. Moreover, as this scFv contains only one lysine residue at the N-terminal, it can be inferred that the PEGylation process is likely site-specific. If multiple PEG moieties had been conjugated to a single scFv molecule, it would have resulted in multiple bands with varying MW [59,60].

### 3.2. Radiolabeling with Technetium Tricarbonyl

The radiolabeling process for both AININ20 and AININ20-PEG with the [^99m^Tc(H_2_O)_3_(CO)_3_] precursor was conducted under mild aqueous conditions at pH 7. According to ITLC results, the labeling led to the production of the desired ^99m^Tc-AININ20 tracer with excellent radiochemical purity in both preparations. RCP of 95.4% and 92.7% was determined for ^99m^Tc-AININ20 and ^99m^Tc-AININ20-PEG, respectively. These high RCP values indicate that the PEGylation process did not significantly impair the ability of the antibody to be efficiently radiolabeled, demonstrating the compatibility of PEGylation with technetium tricarbonyl-based radiochemistry.

### 3.3. In Vitro Stability Studies

The in vitro stability of both ^99m^Tc-AININ20 and ^99m^Tc-AININ20-PEG was quantitatively evaluated in normal saline and human serum at 37 °C for a 24 h period using radio-TLC analysis. In saline, both radiolabeled compounds exhibited high radiochemical purity throughout the incubation period, remaining above 96% at all evaluated time points and up to 24 h. In human serum, a gradual and time-dependent decrease in radiochemical purity was observed for both tracers. Nevertheless, radiochemical purity remained above 95% in the early incubation period in human serum. Notably, the PEGylated antibody consistently demonstrated higher stability compared to the non-PEGylated form, retaining approximately 90% of its initial radiochemical purity at 24 h, whereas ^99m^Tc-AININ20 retained approximately 87%. These results are illustrated in the stability profiles shown in Figure S3. The results indicate that the radiochemical stability of ^99m^Tc-AININ20 and ^99m^Tc-AININ20-PEG in vitro stability in both saline and human serum. Importantly, the PEGylated form consistently maintained its integrity, emphasizing that PEGylation not only preserves but may enhance the stability of the radiolabeled antibody in biological environments.

### 3.4. Logp Analysis

The LogP values for radiolabeled ^99m^Tc-AININ20 and ^99m^Tc-AININ20-PEG were measured via the n-octanol/PBS partition method, yielding values of −1.43 ± 0.05 and −1.38 ± 0.06, respectively. These negative values indicate the hydrophilic nature of the compounds and suggest that crossing the blood–brain barrier via passive diffusion (which typically occurs for lipophilic molecules with LogP > 0) is unlikely [78,79,80]. Nevertheless, imaging data demonstrated the effective presence of the compounds in brain tissue, which may indicate transport via non-passive mechanisms. These findings suggest that, for this class of compounds, the combination of negative LogP and effective brain uptake is indicative of non-passive BBB crossing, although the precise transport mechanism requires further investigation in future studies. These findings suggest that, for this class of compounds, the combination of negative LogP and effective brain uptake is indicative of non-passive BBB crossing, although the precise transport mechanism requires further investigation in future studies. Despite their hydrophilic nature and negative LogP values, both radiotracers showed effective brain accumulation, confirming that AININ20 retains high water solubility even after PEGylation without compromising its brain-targeting capability.

### 3.5. Spect Imaging

**Figure 3:**
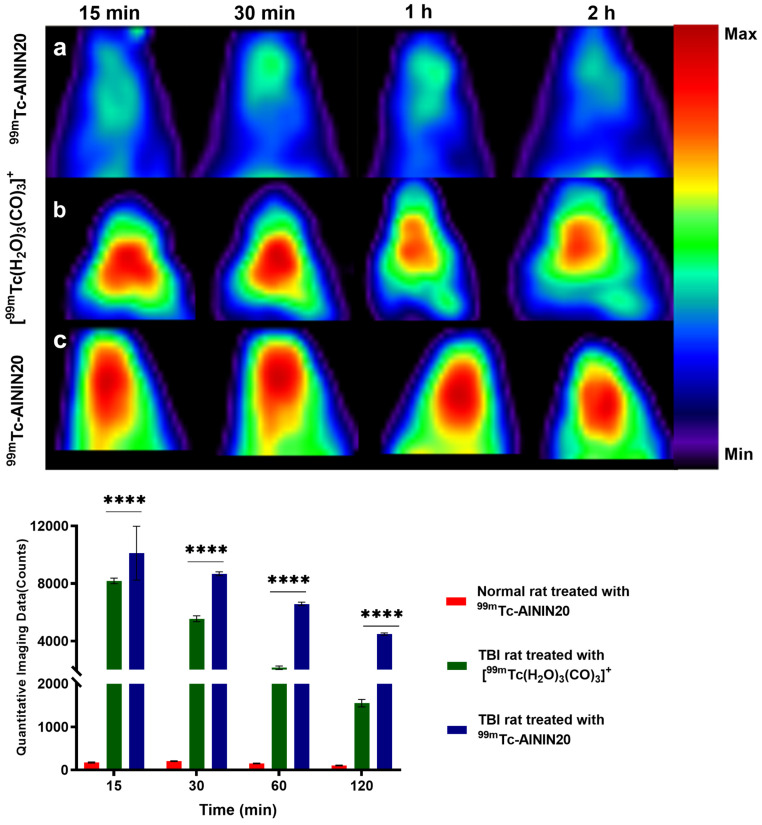
SPECT imaging at 15, 30 min, 1, and 2 h post-injection in different animal models: (**a**) Normal animals treated with ^99m^Tc-AININ20 showed no significant brain signal at any time point, indicating an intact BBB and absence of tauopathy; (**b**) TBI animals treated with ^99m^Tc(CO)_3_ exhibited moderate brain uptake due to BBB disruption; (**c**) TBI animals treated with ^99m^Tc-AININ20 showed significantly higher and sustained brain accumulation across all time points (*p* < 0.0001, ****, 2-way ANOVA with Tukey’s post hoc test, *n* = 3 per group), Data are presented as mean ± SD.

SPECT imaging was performed to evaluate the brain uptake and distribution of the radiolabeled antibodies in TBI rats. The following observations focus on tracer accumulation and retention over different time points post-injection. The 2 h imaging time point was selected to assess the early brain uptake and distribution of the radiolabeled antibodies, providing insights into their initial ability to cross the blood–brain barrier and selectively accumulate in areas affected by TBI-induced pathology. In this study, imaging was performed at 15, 30 min, 1 h, and 2 h post-injection across three groups of animals treated with different approaches, as shown in Figure 3. The first group consisted of normal animals treated with ^99m^Tc-AININ20, which exhibited consistently weak signals at all time points, with a gradual decrease up to 2 h. In healthy animals, no significant brain signal was observed at any time point, indicating that ^99m^Tc-AININ20 was unable to cross the intact BBB in the absence of tauopathy or BBB disruption. The second group, serving as a positive control, included TBI model animals treated solely with the ^99m^Tc(CO)_3_, which showed moderate signal intensity at all time points, reflecting the entry into the brain due to BBB disruption (Figure 3b). However, the observed uptake may remain insufficient for optimal therapeutic or imaging outcomes in longer periods. In contrast, the third group, comprising TBI model animals treated with the ^99m^Tc-AININ20, displayed a significantly higher signal across all time points compared to both control groups, indicating that the antibody’s stronger uptake relies on targeted entry rather than passive diffusion (*p* < 0.0001, Two-way ANOVA followed by Tukey’s multiple comparisons test, *n* = 3).

This substantial increase in signal suggests that the antibody enhances localization and uptake of the radiopharmaceutical in the brain (*p* < 0.0001), emphasizing the efficacy of targeted delivery strategies.

Overall, these findings highlight the crucial role of the antibody in enabling selective delivery across the blood–brain barrier, showcasing its potential to enhance therapeutic and diagnostic outcomes in brain pathologies. These findings can exhibit the selective and specific accumulation of scFv in the brains of TBI model animals, consistent with prior studies highlighting the effectiveness of antibody-based radiopharmaceuticals for targeted CNS delivery under pathological conditions [81].

**Figure 4:**
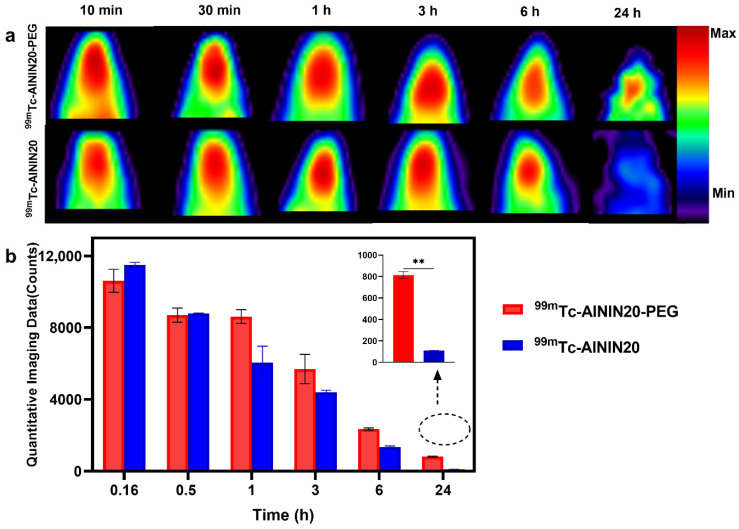
(**a**) SPECT imaging of TBI rat models administered with ^99m^Tc-AININ20 and ^99m^Tc-AININ20-PEG at multiple time points ranging from 10 min to 24 h post-injection. The images illustrate differences in brain uptake and clearance dynamics between the two formulations. (**b**) Quantitative comparison of signal counts over 24 h in TBI animal models. The graph demonstrates the prolonged brain retention of the PEGylated antibody compared to the non-PEGylated version, with a statistically significant difference observed at the 24 h time point (*p* < 0.01, **, 2-way repeated measures ANOVA, *n* = 3 per group), highlighting enhanced stability and sustained localization due to PEGylation. Data are presented as mean ± SD (*n* = 3 per group).

The 24 h imaging time point was selected to monitor the extended retention, biodistribution, and clearance of the radiolabeled antibodies, with a particular focus on comparing the PEGylated and non-PEGylated forms in TBI models. This longer follow-up allowed assessment of whether PEGylation enhances brain retention by improving pharmacokinetics and prolonging the half-life of the radiolabeled antibody. The sustained accumulation observed in the PEGylated group up to 24 h highlights the potential of PEGylation to prolong tracer presence at the target site, supporting its diagnostic advantage for detecting tau pathology in neuroinflammatory conditions like TBI. Figure 4 illustrates the comparative SPECT imaging results over 24 h, demonstrating markedly higher and prolonged brain retention of the PEGylated tracer relative to the non-PEGylated form. In comparing the quantification of the imaging data, it was observed that non-PEGylated scFv had a higher concentration at 10 min post-injection, suggesting rapid initial uptake. By 30 min, the concentration of both antibody forms had reached equilibrium in the brain, after which the concentration decreased. During the first 6 h, the brain imaging profiles of both antibody forms were nearly identical, indicating comparable initial uptake. However, by the 24 h mark, the brain signal of the non-PEGylated antibody had significantly diminished, with no detectable signal remaining. In contrast, the PEGylated antibody continued to exhibit a detectable signal within the brain. These results imply that while PEGylation does not impact the initial brain uptake of the antibody, it appears to overextend its retention time in the brain. This enhancement could be attributed to altered clearance rates or other pharmacokinetic changes introduced by PEGylation [82].

These findings confirm the selective and specific brain accumulation of both AININ20 and AININ20-PEG in the TBI model, which is consistent with previous studies that report PEGylation enhances in vivo stability and prolongs systemic circulation of antibody-based radiotracers, leading to improved imaging contrast and retention in targeted tissues [43,59,83].

### 3.6. In Vivo and Ex Vivo Biodistribution

Ex vivo biodistribution was assessed 2 h post-injection across three groups: healthy controls and TBI models treated with non-PEGylated antibody, and TBI models treated with PEGylated antibody. The ex vivo biodistribution data, presented in Table 1, are consistent with the imaging results across the three animal groups. Specifically, the data revealed no detectable activity in the brains of healthy control rats after being sacrificed (0.047 ± 0.06%ID/g), which matches the imaging findings at 2 h post-injection, showing no signal in healthy control brain tissue. Although Sehlin et al. (2016) targeted amyloid-β rather than tau, their study similarly showed negligible brain uptake of the radiolabeled antibody in healthy controls, supporting our observation that in the absence of pathology, radiotracer accumulation in the brain remains minimal [84]. In contrast, both PEGylated and non-PEGylated formulations exhibited approximately similar levels of brain accumulation in the TBI models. This similarity in brain uptake supports the imaging results, indicating that at 2 h post-injection, both types of radiolabeled AININ20 display comparable distribution patterns in the brains of TBI-affected animals. The absence of a significant signal in normal brain tissue reinforces the specificity of the radiolabeled scFv for pathological conditions.

The high kidney uptake of ^99m^Tc-AININ20 (28.97 ± 1.84%ID/g) reflects its strong renal accumulation, which is typical for small biomolecules. This agrees with the study by Gainkam et al., where high kidney uptake was also observed for two ^99m^Tc-labeled anti-EGFR nanobodies, with uptake increasing further over time [85].

In contrast, the lower kidney accumulation of ^99m^Tc-AININ20-PEG (15.13 ± 1.78%ID/g) underscores the role of PEGylation in reducing renal filtration and prolonging circulation time. The higher liver accumulation of PEGylated scFv at 2 h post-injection (13.36 ± 1.03%ID/g) suggests enhanced hepatic retention, likely due to the reduced renal clearance associated with PEGylation.

Furthermore, uptake in the thyroid was minimal across all groups (ranging from 0.19 ± 0.19 to 0.34 ± 0.84%ID/g), which further supports the high in vivo stability of the radiotracer and confirms negligible free pertechnetate release [68].

**Figure 5:**
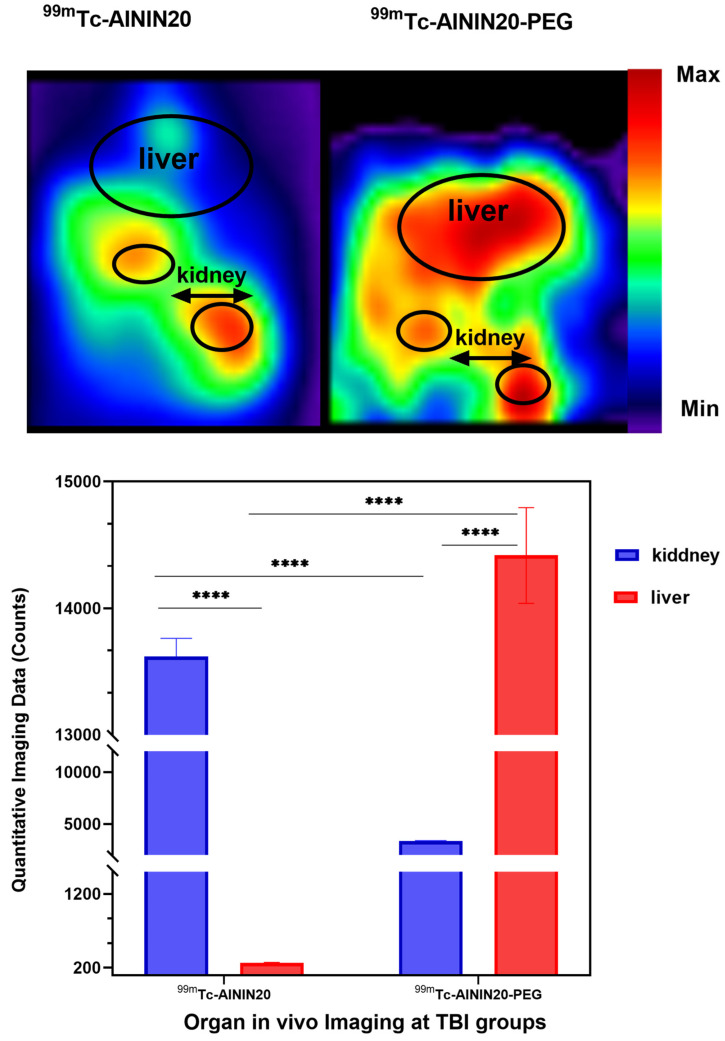
In vivo organ imaging in rats was conducted 24 h after the injection of ^99m^Tc-AININ20 and ^99m^Tc-AININ20-PEG, highlighting the biodistribution of the radiolabeled compounds, particularly in the liver and kidneys (*p* < 0.0001, ****, 2-way ANOVA with Sidak’s multiple comparisons test, *n* = 3 per group). Data are presented as mean ± SD (*n* = 3 per group).

In an in vivo organ imaging study (Figure 5), both 24 h time points following brain imaging were performed using the same animals that received ^99m^Tc-AININ20 and ^99m^Tc-AININ20-PEG. The non-PEGylated formulation showed high kidney uptake, indicating a clearance via renal excretion. This finding is consistent with the general understanding that proteins and antibodies with molecular weights below 60–70 kDa are predominantly eliminated through the kidneys [86], and shorter circulation time, non-PEGylated scFv, facilitates quick renal excretion. However, PEGylation can alter this pathway: even PEG chains up to 20 kDa in size have been shown to reduce renal clearance and shift elimination toward the hepatobiliary system by increasing the overall hydrodynamic size of the conjugate [59,87]. In our study, the PEGylated scFv displayed predominant liver accumulation, supporting previous findings that PEGylation can redirect the excretion route from renal to hepatic pathways.

The integration of ex vivo biodistribution data at 2 h and in vivo imaging data at 24 h provides a comprehensive view of the pharmacokinetics of PEGylated and non-PEGylated scFv. While the ex vivo analysis highlights initial organ-specific distribution, the in vivo imaging reveals long-term distribution and clearance patterns.

The binding of the PEGylated antibody to cis-pT231 tau was directly assessed using ELISA. The results showed a modest reduction in binding (~13%) compared to the non-PEGylated antibody, indicating that the antibody largely retains its specificity (Supplementary Figure S2). In line with these results, follow-up in vivo imaging demonstrated that brain accumulation profiles of the PEGylated and non-PEGylated antibody forms were not significantly different, confirming that target-binding capability was largely preserved in vivo.

Future research should focus on optimizing the PEGylation process to balance efficacy and safety while evaluating its long-term effects in diverse models of tauopathy and other neurodegenerative disorders. Moreover, the development of combination therapies involving scFv and other therapeutic modalities could further enhance treatment outcomes. Our study primarily focused on evaluating the diagnostic potential of PEGylated and non-PEGylated scFv using SPECT imaging in a TBI model characterized by tauopathy. The results indicated that both forms of scFv provided clear imaging of tau aggregates, with PEGylated scFv offering longer circulation times.

Given that the antibody’s specificity for tau pathology has been thoroughly validated in previous studies and documented in the associated patent, in vivo blocking or non-specific antibody controls were not performed in this study. The primary focus of the present work was to evaluate imaging performance and in vivo biodistribution in a TBI model. Experiments to confirm specificity in vivo are planned for future studies.

The enhanced imaging capability of PEGylated scFv observed in our study aligns with the fundamental principles of improved pharmacokinetics through PEGylation, as previously demonstrated in therapeutic contexts. This is particularly significant, given that most prior research on scFv has centered on its therapeutic applications. In contrast to the therapeutic focus of previous research, our findings underscore the potential of PEGylated scFv as a powerful diagnostic tool.

## 4. Conclusions

This study demonstrated the effectiveness of PEGylation in enhancing the pharmacokinetic properties of radiolabeled scFv for imaging and diagnostic applications. Both PEGylated and non-PEGylated scFvs exhibited significantly higher accumulation in TBI models compared to the healthy control group, indicating their potential utility in detecting tau pathology associated with TBI. Quantitative imaging data revealed that PEGylated scFv showed a substantially higher brain concentration 24 h post-administration compared to its non-PEGylated counterpart. This suggests that PEGylation not only enhances the antibody’s brain penetration but also prolongs its presence, offering advantages for long-term imaging and diagnostic accuracy. In contrast, non-PEGylated scFv exhibited faster clearance from the brain, limiting its effectiveness for prolonged diagnostic applications.

Additionally, ex vivo biodistribution studies highlighted distinct clearance patterns, with PEGylated scFv showing preferential hepatic clearance and reduced renal excretion compared to non-PEGylated scFv. These findings underscore the role of PEGylation in modifying drug distribution and clearance, aligning with observed in vivo imaging results.

Overall, PEG-scFv demonstrates significant promise for the early detection and monitoring of tau pathology and neurodegeneration in TBI. Its enhanced pharmacokinetics and prolonged brain retention make it a valuable candidate for both diagnostic and therapeutic applications in neurodegenerative diseases.

## Figures and Tables

**Table 1:** Biodistribution of ^99m^Tc-labeled antibodies (%ID/g) in healthy and TBI Wistar rats 2 h post-injection (Mean ± SD, *n* = 3).

Organ	Healthy Control (^99m^Tc-AININ20)	TBI + Native Ab (^99m^Tc-AININ20)	TBI + PEG-Ab (^99m^Tc-AININ20-PEG)
Brain	0.047 ± 0.11	1.92 ± 0.27	2.12 ± 0.39
Heart	4.28 ± 0.66	3.11 ± 1.615	4.51 ± 0.91
Blood	19.9 ± 0.17	22.56 ± 1.19	24.63 ± 0.18
Liver	2.1 ± 1.13	1.23 ± 1.47	13.36 ± 1.03
Spleen	6.56 ± 0.6	3.803 ± 0.19	3.24 ± 0.61
Kidney	23.09 ± 4.94	28.97 ± 1.84	15.13 ± 1.78
Stomach	3.98 ± 3.13	4.63 ± 1.2	3.49 ± 0.68
Lung	11.44 ± 2.71	12.17 ± 1.143	9.28 ± 1.28
Thyroid	0.34 ± 0.84	0.19 ± 0.19	0.22 ± 0.125
Intestine	7.98 ± 0.74	16.52 ± 0.74	13.97 ± 0.47
Bladder	13.57 ± 0.95	14.03 ± 0.93	8.96 ± 0.61

## Data Availability

The original contributions presented in this study are included in the article/Supplementary Material. Further inquiries can be directed to the corresponding authors.

## Supplementary Materials

The following supporting information can be downloaded at: https://www.mdpi.com/article/10.3390/pharmaceutics18050626/s1, Figure S1: Representative immunofluorescence images from the TBI model. Accumulation of cis-phosphorylated tau (cis-pT231, red) is observed in neurons, with nuclei counterstained by DAPI (blue), confirming pathogenic tau forms in injured brain tissue, consistent with previous studies; Figure S2: The binding affinity of PEGylated and non-PEGylated antibody measured by ELISA. Both antibody forms showed high specificity toward the target antigen, with PEGylation not significantly affecting binding affinity; Figure S3: Radiochemical purity (RCP%) of AININ20 and PEG-AININ20 incubated in saline (S) and human serum (HS) over 24 h at 37 °C. PEGylated formulations exhibited improved stability compared to non-PEGylated counterparts, particularly in human serum.
