# Supplementary material for: Development and Evaluation of “a PEGylated Anti-Tau ScFv for SPECT Imaging” in a Rat Model of Traumatic Brain Injury
Source: Pharmaceutics. 2026 May 20;18(5):626. doi: 10.3390/pharmaceutics18050626 (PMC13210521; doi:10.3390/pharmaceutics18050626)
Supplement: Supplementary file 1 [file pharmaceutics-18-00626-s001.zip › pharmaceutics-4167163-supplementary.pdf]

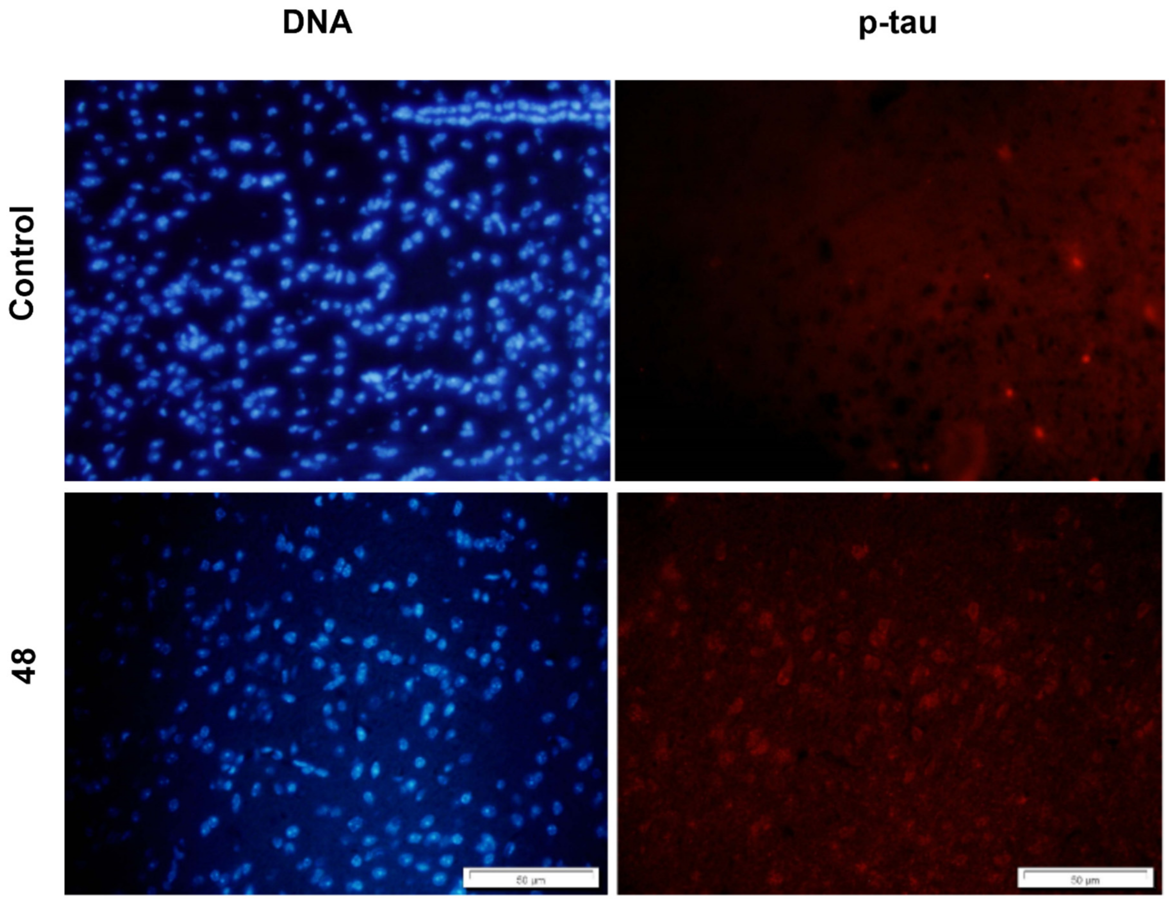

Figure S1. Representative immunofluorescence images from the TBI model. Accumulation of cis-phosphorylated tau (cis-pT231, red) is observed in neurons, with nuclei counterstained by DAPI (blue), confirming pathogenic tau forms in injured brain tissue, consistent with previous studies.

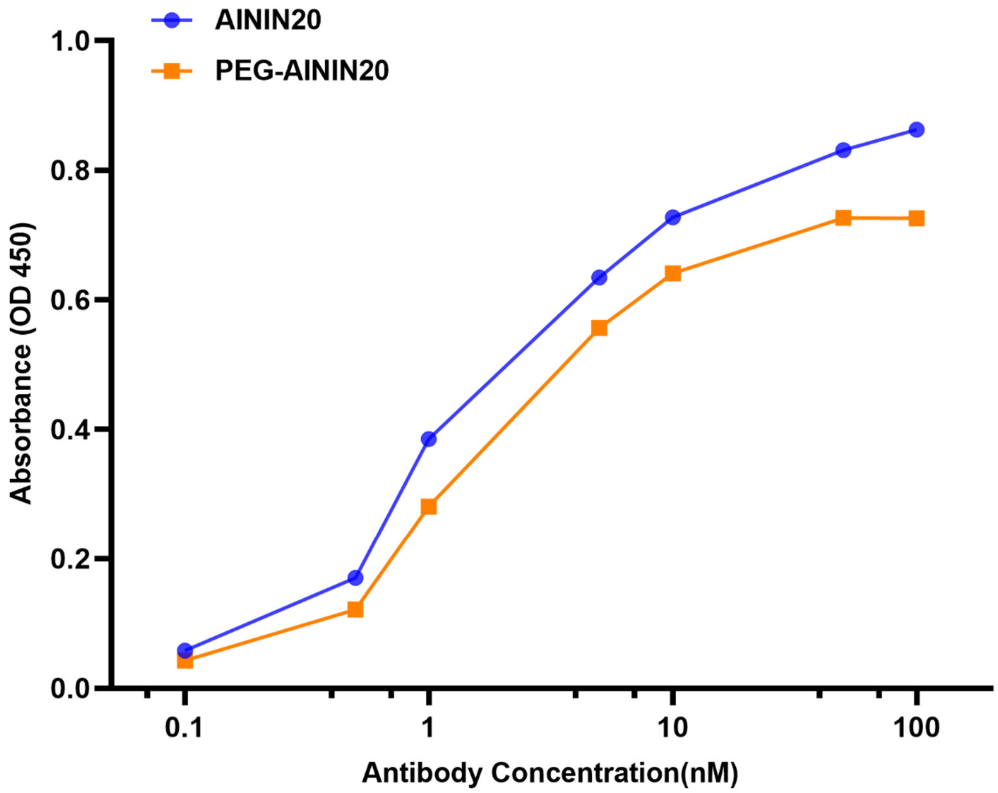

Figure S2. The binding affinity of PEGylated and non-PEGylated antibody measured by ELISA. Both antibody forms showed high specificity toward the target antigen, with PEGylation not significantly affecting binding affinity.

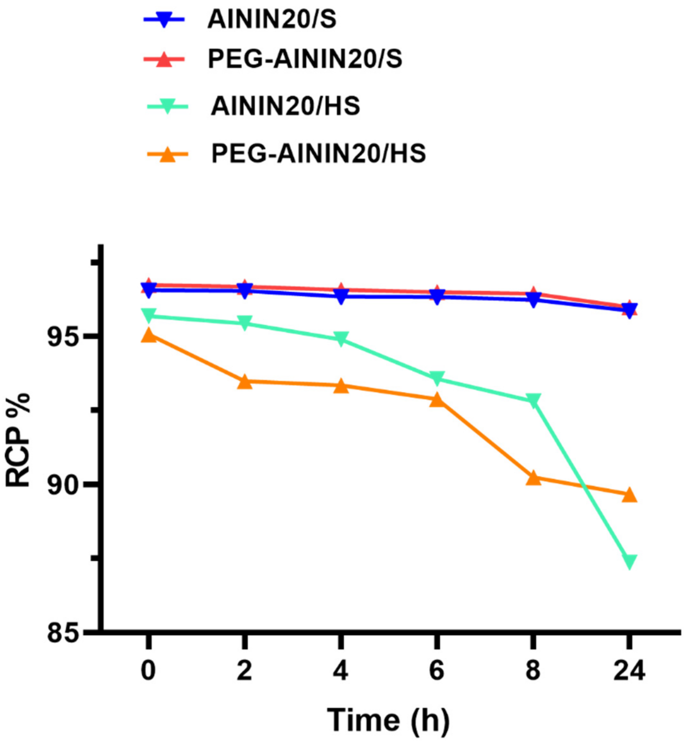

Figure S3. Radiochemical purity (RCP%) of AININ20 and PEG-AININ20 incubated in saline (S) and human serum (HS) over 24 h at 37 °C. PEGylated formulations exhibited improved stability compared to non-PEGylated counterparts, particularly in human serum.
